# Gender disparities in lung cancer incidence in the United States during 2001–2019

**DOI:** 10.1038/s41598-023-39440-8

**Published:** 2023-08-03

**Authors:** Yu Fu, Jun Liu, Yan Chen, Zhuo Liu, Hongbo Xia, Haixia Xu

**Affiliations:** 1 Department of Physical Examination Center, Affiliated Xiaoshan Hospital, Hangzhou Normal University, Hanghzou, China; 2 Department of Clinical Laboratory, Affiliated Xiaoshan Hospital, Hangzhou Normal University, Hanghzou, 311202 China; 3 Department of Gastroenterology, PLA Strategic Support Force Characteristic Medical Center, Beijing, China; 4 Department of Respiratory Therapy, Affiliated Xiaoshan Hospital, Hangzhou Normal University, Hanghzou, China

**Keywords:** Cancer, Risk factors

## Abstract

Lung cancer ranks as one of the top malignancies and the leading cause of cancer death in both males and females in the US. Using a cancer database covering the entire population, this study was to determine the gender disparities in lung cancer incidence during 2001–2019. Cancer patients were obtained from the National Program of Cancer Registries (NPCR) and Surveillance, Epidemiology and End Results (SEER) database. The SEER*Stat software was applied to calculate the age-adjusted incidence rates (AAIR). Temporal changes in lung cancer incidence were analyzed by the Joinpoint software. A total of 4,086,432 patients (53.3% of males) were diagnosed with lung cancer. Among them, 52.1% were 70 years or older, 82.7% non-Hispanic white, 39.7% from the South, and 72.6% non-small cell lung cancer (NSCLC). The AAIR of lung cancer continuously reduced from 91.0 per 100000 to 59.2 in males during the study period, while it increased from 55.0 in 2001 to 56.8 in 2006 in females, then decreased to 48.1 in 2019. The female to male incidence rate ratio of lung cancer continuously increased from 2001 to 2019. Gender disparities were observed among age groups, races, and histological types. In those aged 0–54 years, females had higher overall incidence rates of lung cancer than males in recent years, which was observed in all races (except non-Hispanic black), all regions, and adenocarcinoma and small cell (but not squamous cell). Non-Hispanic black females aged 0–54 years had a faster decline rate than males since 2013. API females demonstrated an increased trend during the study period. Lung cancer incidence continues to decrease with gender disparities among age groups, races, regions, and histological types. Continuous anti-smoking programs plus reduction of related risk factors are necessary to lower lung cancer incidence further.

## Introduction

Lung cancer currently ranks as the second most common malignancy and the leading cause of cancer death in both males and females. It is estimated that lung cancer will be diagnosed in approximately 238,340 individuals and cause 127,070 deaths in the US in 2023^1^. More efforts are needed to identify the risk factors and develop effective approaches to curb the incidence and mortality of the disease. Studies on gender disparity may help to better understand the underlying etiology for lung cancer development.

Previous studies have shown the gender disparity in lung cancer incidence. The age standardized incidence rate of lung cancer was generally higher in males than in females by race and histological types^2–7^. Temporally, males had continuous and steeper declines in lung cancer^6–8^, whereas females displayed increased lung cancer incidence before the year 2006–2007 and then decreased incidence thereafter^6,7^. A more recent study reported reduced incidence rates of lung cancer in males by 2.6% per year and in females by 1.1% per year since 2006^1^. Among those aged 30 to 54 years, the incidence of lung cancer generally decreased in both males and females, but females displayed a higher incidence than males only in whites and Hispanics during 2010–2014^8^.

It is noted that most previous studies reported the gender disparity in lung cancer incidence in 2016 or earlier. The gender disparities in lung cancer by age, race, region, and histological type, particularly in those aged 0–54, have not been updated. Databases used in most previous studies only included a portion of the US population. Using a database capturing the entire population, this study was to determine the gender difference in lung cancer incidence in all patients and those aged 0–54 among races, regions, and histological types during 2001–2019. This information is essential for unraveling the etiologic causes and developing targeted prevention.

## Patients and methods

We acquired lung cancer patients diagnosed during 2001–2019 from the National Program of Cancer Registries (NPCR) and Surveillance, Epidemiology and End Results (SEER) Program SEER*Stat Database^9^. This database includes 100% of the population in the US, with an exception in a couple of years (https://www.cdc.gov/cancer/uscs/technical_notes/criteria/registries.htm). Based on the International Classification of Diseases for Oncology, Third Edition (ICD-O-3), we categorized lung cancer histological types of small cell lung cancer (8002 and 8041–5), non-small cell lung cancer (NSCLC) and others. The NSCLC was further classified as adenocarcinoma (8140, 8211, 8230–8231, 8250–8260, 8323, 8480–8490, 8550–8551, 8570–8574 and 8576), squamous cell (8051–2, 8070–6, 8078, 8083–4, 8090, 8094, 8120 and 8123) and other NSCLC^2^. The races in the database were divided as non-Hispanic white (NHW), non-Hispanic black (NHB), Hispanic, non-Hispanic American Indian and Alaska Native (AIAN), and non-Hispanic Asian or Pacific Islanders (API). The US is divided into four statistical regions by the Census Burea: Northeast, Midwest, South, and West (https://www2.census.gov/geo/pdfs/maps-data/maps/reference/us_regdiv.pdf). Because patients’ information in the database is de-identified, human investigation approval was waived for this current study.

### Statistical analysis

We used the Chi Square test to analyze categorical data. The age-adjusted incidence rate (AAIR) per 100,000 patients was calculated using the software SEER*Stat version 8.4.1 (National Cancer Institute) and the 2000 US standard population. The confidence interval (CI) was calculated using Tiwari et al.'s 2006 modifications^10^. The Joinpoint Regression Program (version 4.9.1.0, the National Cancer Institute, USA) was applied to generate incidence graphs and calculate the annual percent change (APC) and the average annual percentage change (AAPC) using the least square method. For all analyses, *p* < 0.05 was deemed statistically significant.

## Results

### Characteristics of lung cancer patients diagnosed during 2001–2019.

Lung cancer was diagnosed in 4086435 patients, with 53.3% of males during the study period (Table 1). As high as 52.1% of patients were at ages 70 years or older (33.0% for 70–79 years and 19.1% for 80 years and over). Among them, 82.7% were NHW, and 10.2% were NHB. The South region had the highest number and proportion (39.7%) of lung cancer cases. Histologically, small cell and NSCLC comprised 12.9% and 72.6% of all lung cancer, respectively.

**Table 1:** Characteristics of lung cancer patients by gender diagnosed during 2001–2019.

Variable	Total n (%)	Male n (%)	Female n (%)	*P* value
	4,086,435 (100)	2,178,990 (53.3)	1,907,445 (46.7)	
Age (years)				
0–39	23,029 (0.6)	11,128 (48.3)	11,901 (51.7)	< 0.0001
40–54	368,290 (9.0)	189,151 (51.4)	179,139 (48.6)	
55–69	1,565,663 (38.3)	860,315 (54.9)	705,348 (45.1)	
70–79	1,347,684 (33.0)	723,624 (53.7)	624,060 (46.3)	
80 and over	781,769 (19.1)	394,772 (50.5)	386,997 (49.5)	
Race				
NHW	3,379,097 (82.7)	1,784,158 (52.8)	1,594,939 (47.2)	< 0.0001
NHB	418,212 (10.2)	235,580 (56.3)	182,632 (43.7)	
Hispanic	163,576 (4.0)	90,554 (55.4)	73,022 (44.6)	
API	93,683 (2.3)	51,880 (55.4)	41,803 (44.6)	
AIAN	21,338 (0.5)	10,880 (51.0)	10,458 (49.0)	
Region				
Northeast	796,586 (19.5)	403,582 (50.7)	393,004 (49.3)	< 0.0001
Midwest	978,255 (23.9)	522,055 (53.4)	456,200 (46.6)	
South	1,621,560 (39.7)	898,721 (55.4)	722,839 (44.6)	
West	690,034 (16.9)	354,632 (51.4)	335,402 (48.6)	
Histology				
Small cell	525,403 (12.9)	262,550 (50.0)	262,853 (50.0)	< 0.0001
NSCLC	296,8334 (72.6)	1,604,429 (54.1)	1,363,905 (45.9)	
Squamous cell	1,462,029 (35.8)	711,986 (48.7)	750,043 (51.3)	
Adenocarcinoma	838,669 (20.5)	529,416 (63.1)	309,253 (36.9)	
Other NSCLC	667,636 (16.3)	363,027 (54.4)	304,609 (45.6)	
Other	592,698 (14.5)	312,011 (52.6)	280,687 (47.4)	

*NSCLC* non-small cell lung cancer.

Though more males were diagnosed with lung cancer cases than females in all patients, females had a slightly higher proportion of lung cancer at young ages (50.8% in the 0–39 year age group). Among all races, AIAN had the highest proportion of female patients (49.0%). In all regions, The Northeast region had the highest proportion of female patients (49.3%). Females (51.3% vs. 48.7% males) were more likely to be diagnosed with lung adenocarcinoma (Table 1).

### Gender disparity in overall age adjusted incidence rate of lung cancer and its trend during 2001–2019

**Figure 1:**
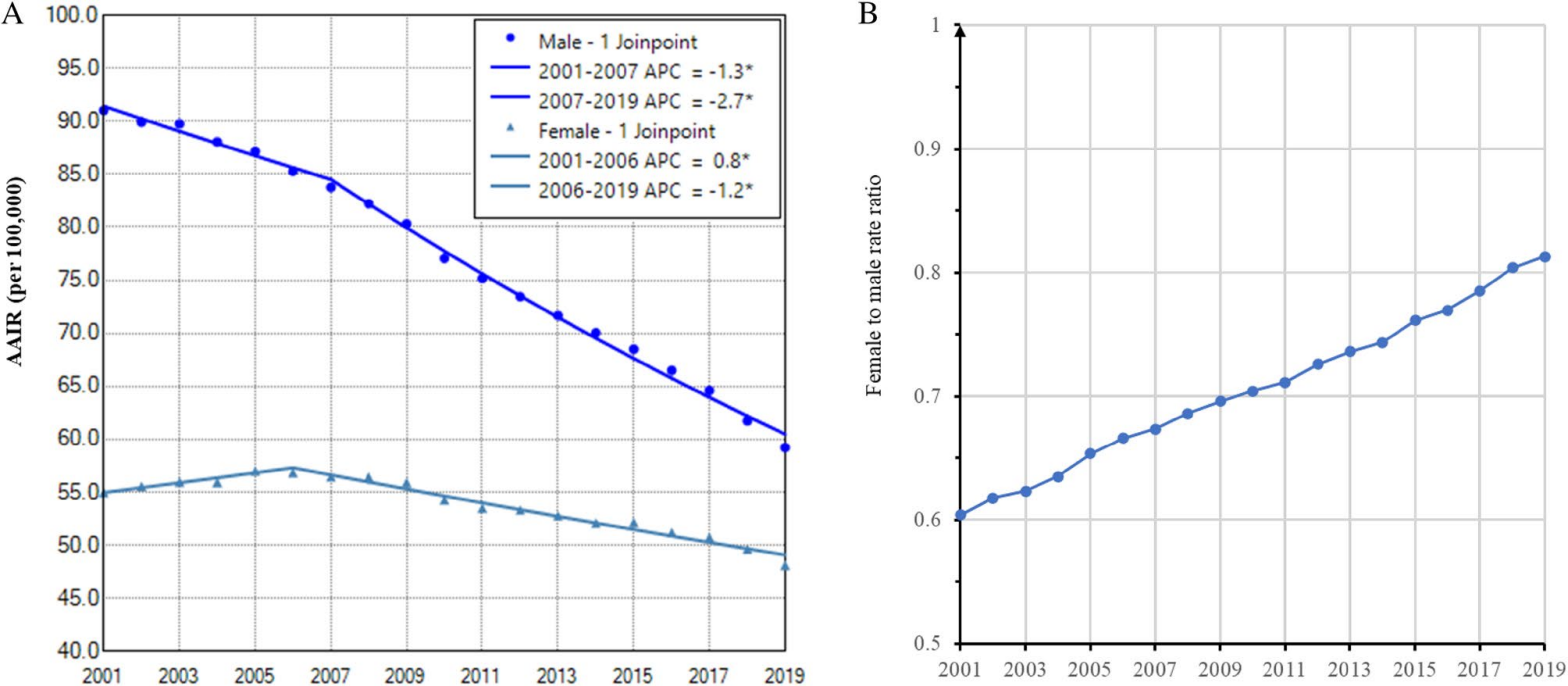
Temporal change of lung cancer incidence by gender during 2001–2019. (**A**). Change in AAIR by gender. (**B**). Female to male rate ratios of lung cancer incidence rate. AAIR: age adjusted incidence rate; APC: annual percentage change. *Indicates a significant change in APC.

The AAIR of lung cancer was higher in males during the entire period (Fig. 1A). Lung cancer incidence in males decreased faster from 91.0 per 100,000 in 2001 to 59.2 in 2019. The APCs were − 1.3 (95% confidence interval (CI): − 1.8 to − 0.8, *P* < 0.001) from 2001–2007 and − 2.7 (95% CI: − 2.9 to − 2.6, *P* < 0.001) from 2007 to 2019. In contrast, lung cancer incidence in females increased from 55.0 per 100,000 to 56.8 with an APC of 0.8 (95% CI: 0.2 to 1.5, *P* = 0.014) during 2001–2006, and then declined to 48.1 in 2019, with an APC of − 1.2 (95% CI: − 1.3 to − 1.0, *P* < 0.001) (Fig. 1A and Table 2). The AAPCs were − 2.3 (95% CI: − 2.4 to − 2.1, *P* < 0.001) for males and − 0.6 (95% CI: − 0.8 to − 0.4, *P* < 0.001) for females. Accordingly, we observed a continuous increase in the female to male incidence rate ratio of lung cancer (Fig. 1B) and the proportion of female cases from 2001 to 2019 (Supplemental data Table S1).

**Table 2:** Trends of age adjusted incidence rates of lung cancer during 2001–2019.

Groups	Trend 1	APC	95% CI	*P* value	Trend 2	APC	95% CI	*P* value	Trend 3	APC	95% CI	*P* value
Gender												
Male	2001–2007	− 1.3*	− 1.8 to − 0.8	< 0.001	2007–2019	− 2.7*	− 2.9 to − 2.6	< 0.001				
Female	2001–2006	0.8*	0.2 to 1.5	0.014	2006–2017	− 1.2*	− 1.3 to − 1.0	< 0.001				
Race												
NHW												
Male	2001–2007	− 1.1*	− 1.6 to − 0.6	< 0.001	2007–2019	− 2.6*	− 2.7 to − 2.4	< 0.001				
Female	2001–2006	1.1*	0.3 to 1.8	0.008	2006–2019	− 1.0*	− 1.1 to − 0.8	< 0.001				
NHB												
Male	2001–2009	− 1.9*	− 2.3 to − 1.5	< 0.001	2009–2019	− 3.4*	− 3.7 to − 3.1	< 0.001				
Female	2001–2009	0.3	− 0.2 to 0.7	0.285	2009–2019	− 1.6*	− 1.9 to -1.3	< 0.001				
Hispanic												
Male	2001–2010	− 1.1*	− 1.8 to − 0.4	0.003	2010–2019	− 2.2*	− 2.7 to − 1.7	< 0.001				
Female	Female	0.51	− 0.1 to 1.1	0.099	2009–2019	− 0.4*	-0.7 to 0.0	0.041				
AIAN												
Male	2001–2013	0.2	− 0.9 to 1.3	0.680	2013–2019	− 4.3*	− 6.7 to − 1.8	0.002				
Female	2001–2017	0.7*	0.1 to 1.2	0.020	2017–2019	− 7.3	− 17.3 to 3.9	0.175				
API												
Male	2001–2005	− 1.6	− 3.5 to 0.4	0.115	2005–2019	− 3.0*	− 3.3 to − 2.8	< 0.001				
Female	2001–2007	0.1	− 1.0 to 1.1	0.909	2007–2019	− 1.1*	− 1.4 to − 0.8	< 0.001				
Region												
West												
Male	2001–2009	− 2.2*	− 2.6 to − 1.9	< 0.001	2009–2019	− 3.6*	− 3.8 to − 3.3	< 0.001				
Female	2001–2006	− 0.5	− 1.1 to 0.2	0.144	2006–2017	− 2.0*	− 2.2 to − 1.8	< 0.001	2017–2019	− 4.1*	− 6.8 to − 1.2	0.009
South												
Male	2001–2005	− 0.4	− 1.5 to 0.7	0.442	2005–2019	− 2.8*	− 3 to − 2.6	< 0.001				
Female	2001–2005	1.4*	0.4 to 2.5	0.012	2005–2019	− 1.2*	− 1.3 to − 1.1	< 0.001				
Northeast												
Male	2001–2007	− 1.1*	− 1.6 to − 0.6	0.001	2007–2019	− 2.3*	− 2.5 to − 2.1	< 0.001				
Female	2001–2007	1.0*	0.2 to 1.7	0.013	2007–2019	− 0.9*	− 1.1 to -0.6	< 0.001				
Midwest												
Male	2001–2017	− 1.8*	− 2.0 to − 1.7	< 0.001	2017–2019	− 5.5*	− 9.0 to − 1.8	0.007				
Female	2001–2007	1.1*	0.4 to 1.8	0.005	2007–2019	− 0.7*	− 0.9 to − 0.5	< 0.001				
Histology												
Small cell												
Male	2001–2006	− 2.4*	− 2.9 to − 1.9	< 0.001	2006–2019	− 3.2*	− 3.4 to − 3.1	< 0.001				
Female	2001–2007	− 1.1*	− 1.6 to − 0.6	< 0.001	2007–2019	− 2.2*	− 2.4 to − 2	< 0.001				
Adenocarcinoma												
Male	2001–2015	1.1*	0.7 to 1.4	< 0.001	2015–2019	− 4.7*	− 6.6 to − 2.6	< 0.001				
Female	2001–2015	2.5*	2.2 to 2.9	< 0.001	2015–2019	− 4.1*	− 5.9 to − 2.2	< 0.001				
Squamous cell												
Male	2001–2005	− 2.7*	− 3.5 to − 1.9	< 0.001	2005–2012	0.3	− 0.2 to 0.8	0.187	2012–2019	− 3.5*	− 3.8 to − 3.1	< 0.001
Female	2001–2004	− 0.1	− 2.2 to 2.1	0.916	2004–2012	1.7*	1.2 to 2.3	< 0.001	2012–2019	− 1.9*	− 2.4 to − 1.5	< 0.001

*APC* annual percentage change. *AIAN* Non-Hispanic American Indian and Alaska Native; *API* non-Hispanic Asian or Pacific Islander; *CI* confidence interval; *NHB* non-Hispanic black; *NHW* non-Hispanic white.

*Indicates a significant change of APCs.

### Gender difference in age adjusted incidence rate of lung cancer among age groups

**Figure 2:**
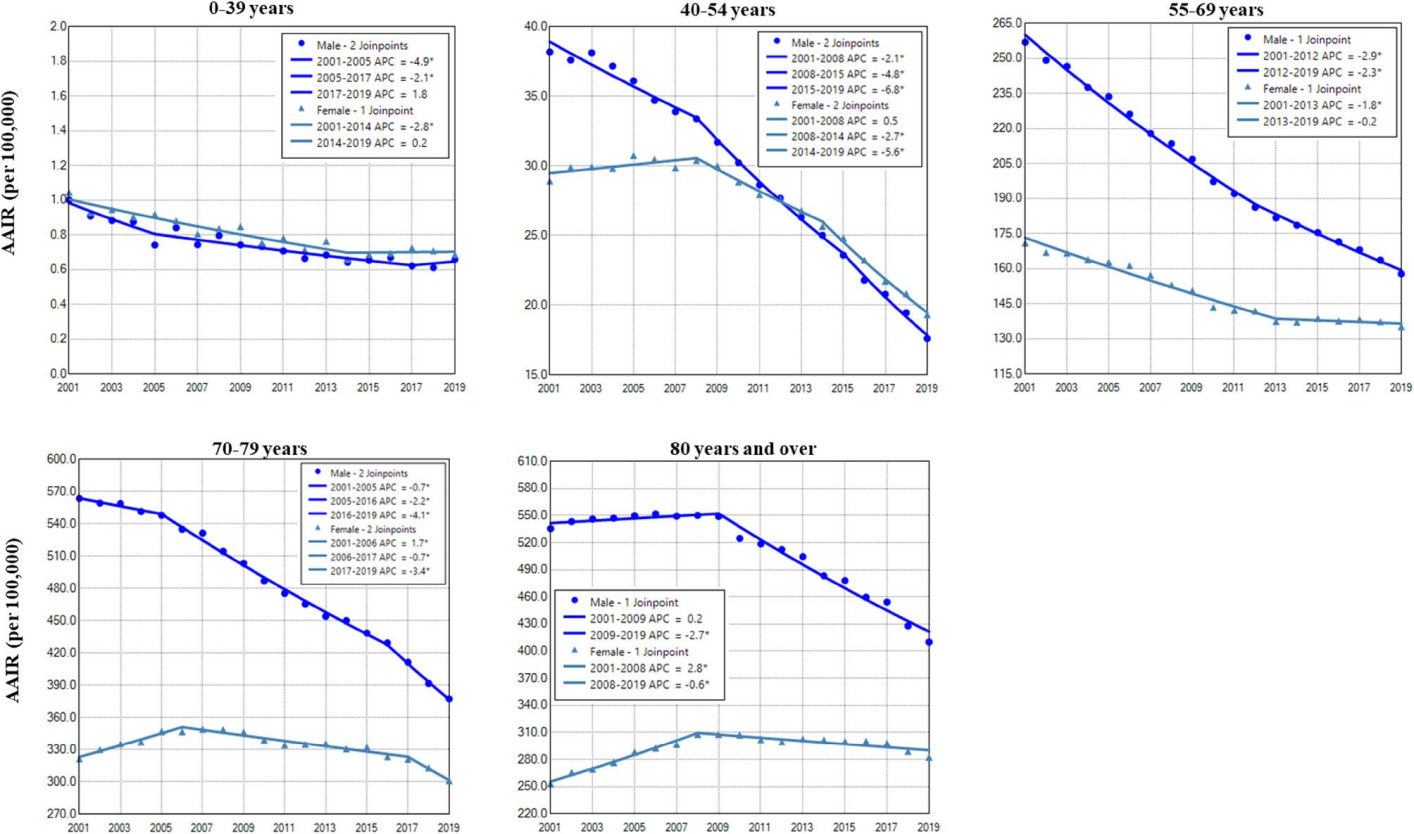
Temporal change of age adjusted incidence rate (AAIR) of lung cancer in males and females by age group during 2001–2019. AAIR: age adjusted incidence rate; APC: annual percentage change. *Indicates a significant change.

Females had a higher incidence rate of lung cancer than males in those aged 0–34 years during 2001–2019 and in 40–54 years during 2013–2019. In contrast, males had a higher incidence of lung cancer than females in older age groups during the study period (Fig. 2 and Table 2). Except for both males and females aged 0–39 years, and females aged 55–69 years, males and females in other age groups displayed continuously decreased incidences of lung cancer with a faster rate in males in the entire period or recent years (Fig. 2 and Table 2). There was a trend of decreased female to male rate ratios in older age groups (Supplemental Data Figure S1).

### Gender difference in age adjusted incidence rate of lung cancer among races

**Figure 3:**
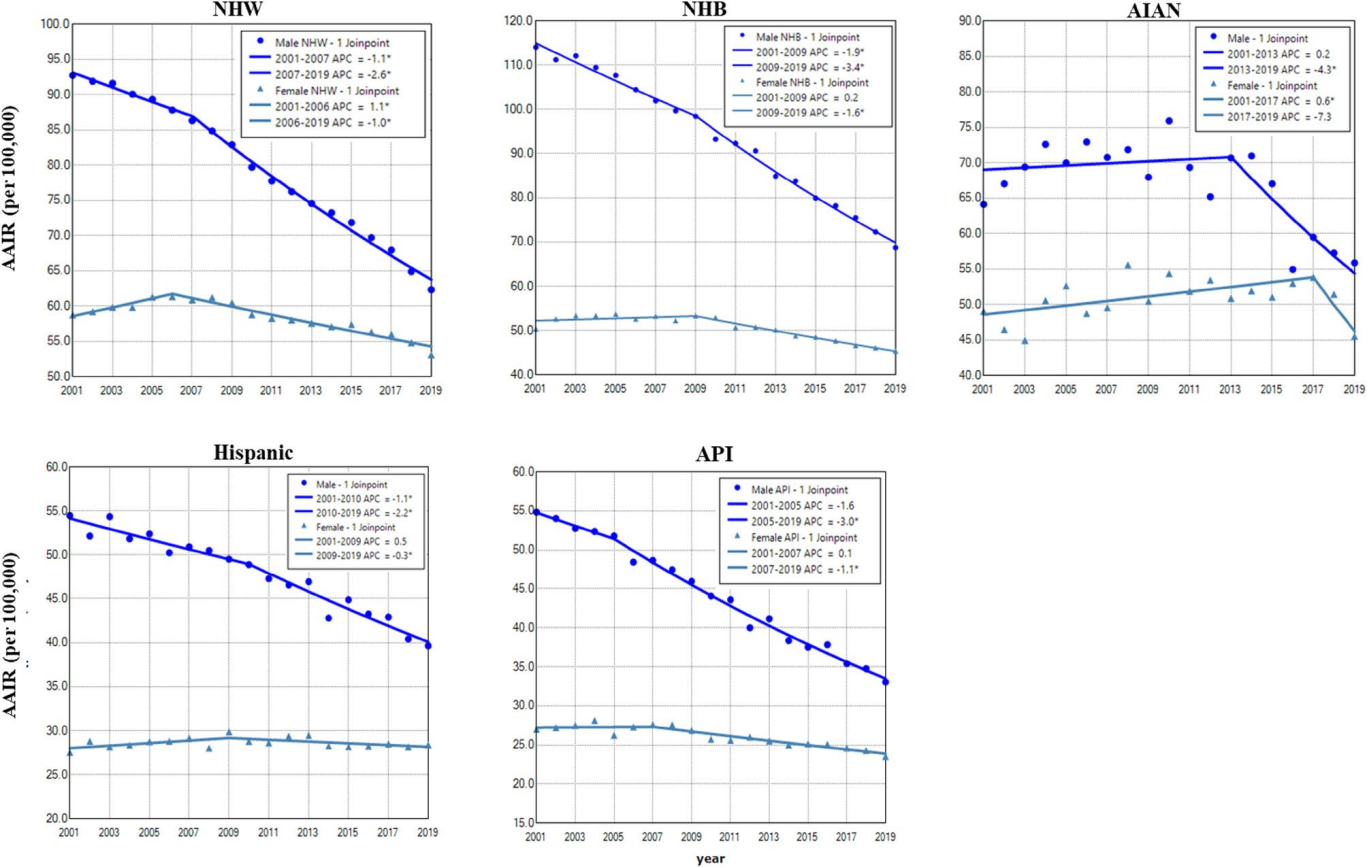
Temporal change of age adjusted incidence rate of lung cancer in males and females by race during 2001–2019. AAIR: age adjusted incidence rate; APC: annual percentage change. AIAN: non-Hispanic American Indian and Alaska Native; API: non-Hispanic Asian or Pacific Islander; NHB: non-Hispanic black; NHW: non-Hispanic white. *Indicates a significant change.

Among all races, NHB and NHW had the highest incidence in males and females, respectively. Hispanic and API males and females had the lowest incidence rates of lung cancer during the period (Fig. 3). Except for AIAN, males in other races had displayed continuous decreases in lung cancer incidence during the entire period. Females of all races had increased or stabilized incidences in earlier years, then decreased incidences in recent years. Males had a faster rate of decrease than females among all races, and NHB males had the most rapid decline among all races (Fig. 3 and Table 2). NHB had the lowest female to male rate ratio (Supplemental Data Figure S2).

### Gender difference in age adjusted incidence and trend of lung cancer among regions

**Figure 4:**
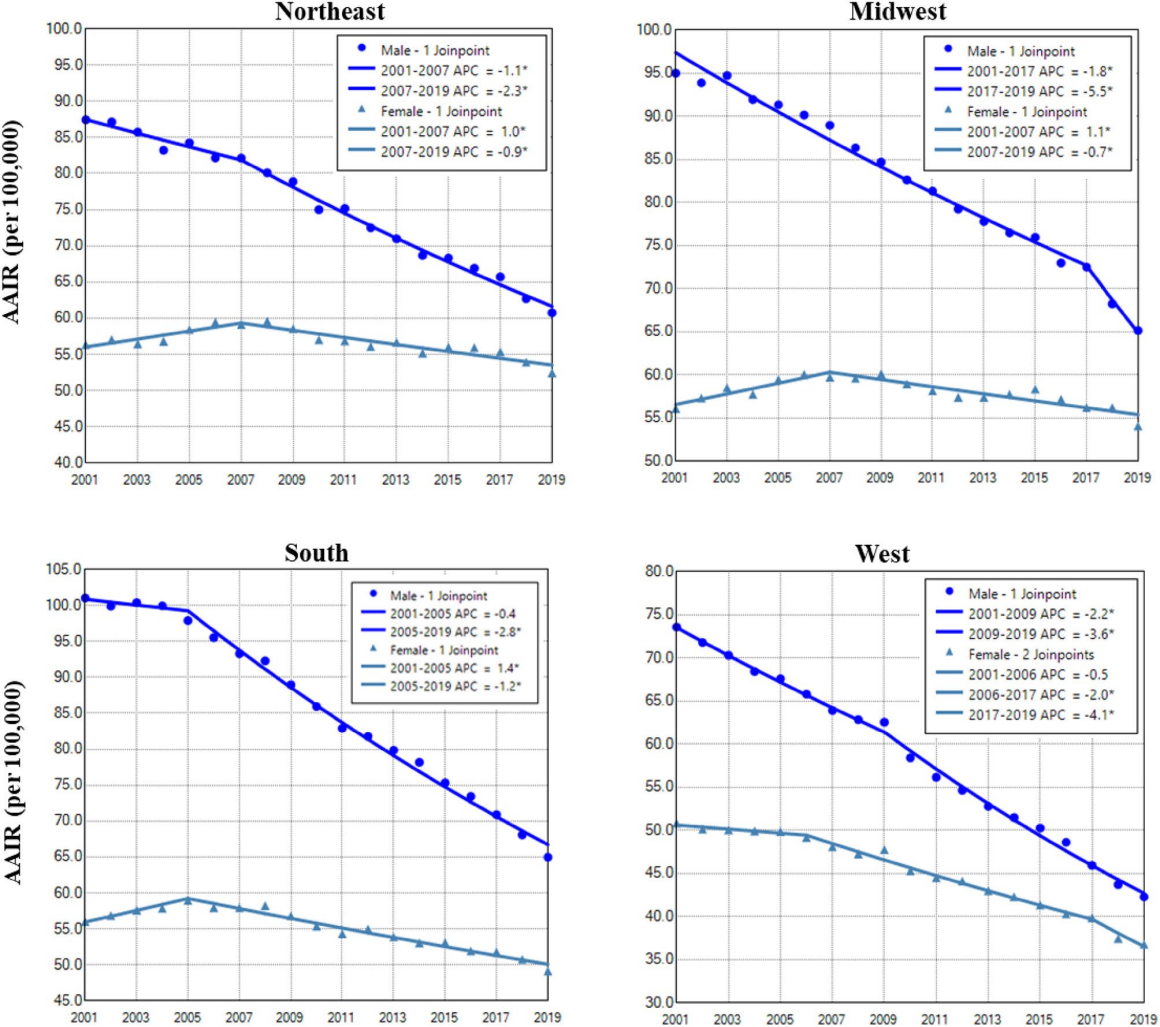
Temporal change of age adjusted incidence of lung cancer in males and females by region during 2001–2019. AAIR: age adjusted incidence rate; APC: annual percentage change. *Indicates a significant change in APC.

Males in the South region had the highest AAIR of lung cancer, whereas the West region had the lowest incidence in males and females (Fig. 4). Males in all regions had continuous decreases in lung cancer incidences in the entire or nearly the whole period. Females in all regions had decreased trend only in recent years (Fig. 4 and Table 2). The West region had the highest female to male rate ratio, whereas the South region had the lowest rate ratio (Supplemental Data Figure S3).

### Gender difference in age adjusted incidence and trend of lung cancer among histological types

**Figure 5:**
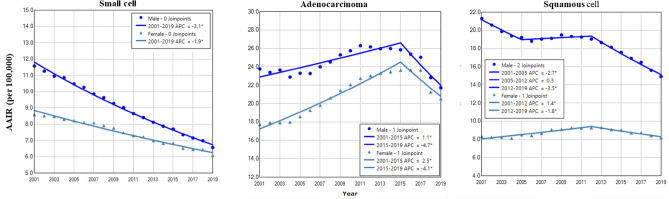
Temporal change of age adjusted incidence of lung cancer in males and females by histological type during 2001–2019. AAIR: age adjusted incidence rate; APC: annual percentage change. *Indicates a significant change in APC.

Adenocarcinoma had the highest incidence rate in both males and females (Fig. 5). The incidence of small cell showed a trend of continuous decline in males and females, with a faster rate in males during 2001–2019 (Fig. 5 and Table 2). Adenocarcinoma in males and females had increased incidence during 2001–2015 with a faster rate in females, and then displayed decreased incidence during 2015–2019 (Fig. 5 and Table 2). Squamous cell incidence in males decreased from 2001 to 2005, stabilized during 2005–2012 and declined between 2012 and 2019 (Fig. 5 and Table 2). The female to male rate ratio was the lowest in squamous cell lung cancer (Supplemental Data Figure S4).

### Gender disparity in lung cancer among those aged 0–54 years

**Figure 6:**
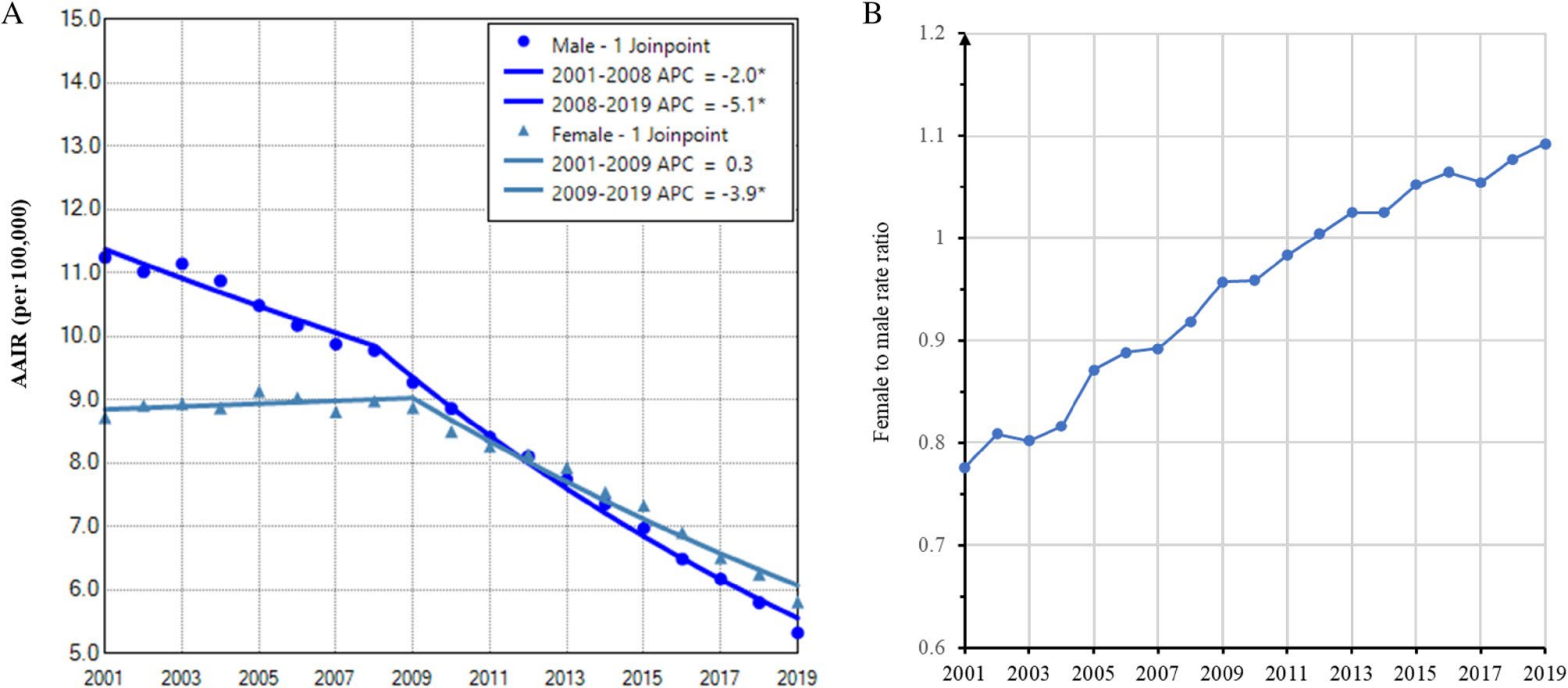
Temporal change of age adjusted incidence of lung cancer by gender in those aged 0–54 years during 2001–2019. (**A**). Change in AAIR by gender. (**B**). Female to male rate ratio of lung cancer incidence rate. AAIR: age adjusted incidence rate; APC: annual percentage change. *Indicates a significant change in APC.

Generally, NHW had a lower proportion in this age group (73.2%) compared to that in all age groups (82.7%) (Table 3). The South region still had the highest proportion of cases. As high as 42% of patients had lung adenocarcinoma. There were higher proportions of female cases in all races except NHB, in Northeast and West regions, and in small cell and adenocarcinoma (Table 3). Temporally, males still had continuously and more rapidly decreased incidences in the study period, while females showed stabilized incidence between 2001 and 2009, then declined incidence between 2009 and 2019 (Fig. 6A). The female to male rate ratio increased from 0.8 in 2001 to 1.1 in 2019, reaching over 1.0 since 2012 (Fig. 6B).

**Table 3:** Characteristics of lung cancer patients of 0–54 years old diagnosed during 2001–2009.

Variable	Total n (%)	Male n (%)	Female n (%)	*P* value
	391,319 (100)	200,279 (51.2)	191,040 (48.8)	
Race				
NHW	124,667 (73.2)	60,385 (48.4)	64,282 (51.6)	< 0.0001
NHB	26,732 (15.7)	14,201 (53.1)	12,531 (46.9)	
Hispanic	10,825 (6.4)	5231 (48.3)	5594 (51.7)	
API	6267 (3.7)	2964 (47.3)	3303 (52.7)	
AIAN	1130 (0.7)	501 (44.3)	629 (55.7)	
Region				
Northeast	75,417 (19.3)	36,361 (48.2)	39,056 (51.8)	< 0.0001
Midwest	96,011 (24.5)	48,869 (50.9)	47,142 (49.1)	
South	164,593 (42.1)	87,665 (53.3)	76,928 (46.7)	
West	55,298 (14.1)	27,384 (49.5)	27,914 (50.5)	
Histology				
Small cell	56,193 (14.4)	27,692 (49.3)	28,501 (50.7)	< 0.0001
NSCLC	303,892 (77.7)	15,4487 (50.8)	149,405 (49.2)	
Adenocarcinoma	162,493 (41.5)	73,125 (45.0)	89,368 (55.0)	
Squamous cell	55,501 (14.2)	37,034 (66.7)	18,467 (33.3)	
Other NSCLC	85,898 (22.0)	44,328 (51.6)	41,570 (48.4)	
Other	31,234 (8.0)	18,100 (57.9)	13,134 (42.1)	

*NSCLC* non-small cell lung cancer.

**Figure 7:**
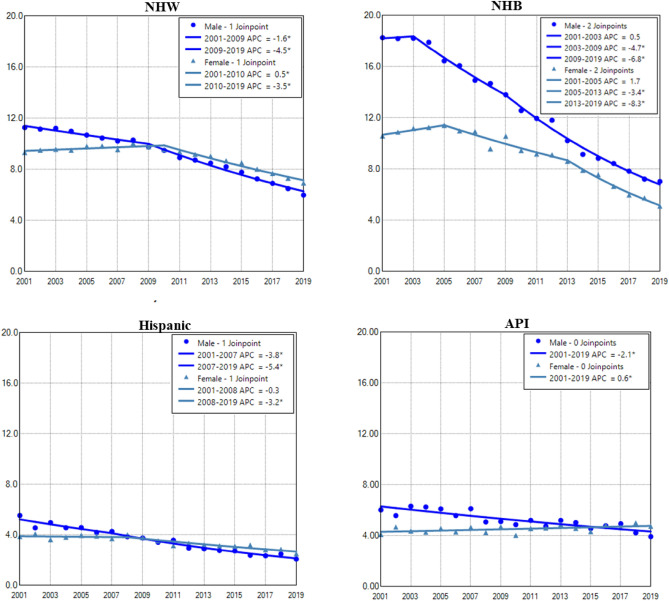
Temporal change of age adjusted incidence of lung cancer in males and females aged 0–54 years by race during 2001–2019. AAIR, age adjusted incidence rate. APC, annual percentage change. *Indicates a significant change in APC.

Except for NHB, females of other races had a higher incidence of lung cancer in recent years (Fig. 7 and Supplemental Data Figure S5). Males in other races still had a faster and continuous decrease in lung cancer. In contrast, NHB females declined more rapidly during 2013–2019, whereas API females slowly increased from 2001 to 2019 (Fig. 7 and Table 4).

**Table 4:** Trend of age adjusted incidence rates of lung cancer in those aged 0–54 years.

Groups	Trend 1	APC	95% CI	*P* value	Trend 2	APC	95% CI	*P* value	Trend 3	APC	95% CI	*P* value
Gender												
Male	2001–2008	− 2.0*	− 2.6 to − 1.4	< 0.001	2008–2019	− 5.1*	− 5.4 to − 4.7	< 0.001				
Female	2001–2009	0.3	− 0.4 to 0.9	0.410	2009–2019	− 3.9*	–4.4 to –3.4	< 0.001				
Race												
NHW												
Male	2001–2009	− 1.6*	− 2.2 to − 1.1	< 0.001	2009–2019	− 4.5*	− 5 to − 4.1	< 0.001				
Female	2001–2010	0.5*	0 to 1.0	0.042	2010–2019	− 3.5*	− 4.0 to − 3.0	< 0.001				
NHB												
Male	2001–2003	0.5	− 7.3 to 8.9	0.904	2003–2009	–4.7*	− 6.4 to –3	< 0.001	2009–2019	− 6.8*	− 7.6 to − 6.1	< 0.001
Female	2001–2005	1.7	− 1.6 to 5.2	0.286	2005–2013	− 3.4*	− 4.7 to − 2	< 0.001	2013–2019	− 8.3*	− 10.3 to − 6.3	< 0.001
Hispanic												
Male	2001–2007	− 3.8*	− 6.4 to − 1.2	0.007	2007–2019	− 5.4*	− 6.3 to − 4.4	< 0.001				
Female	2001–2008	− 0.3	− 2.3 to 1.8	0.793	2008–2019	–3.2*	− 4.1 to − 2.3	< 0.001				
AIAN												
Male	2001–2004	11.0	− 9.5 to 36.0	0.293	2004–2019	− 2.1*	− 3.7 to − 0.5	0.014				
Female	2001–2009	5.1*	0.2 to 10.2	0.041	2009–2019	− 2.3	− 5.3 to 0.8	0.140				
API												
Male	2001–2019	–2.1*	− 2.6 to − 1.5	< 0.001								
Female	2001–2019	0.6*	0.1 to 1.0	0.015								
Region												
West												
Male	2001–2008	− 3.4*	− 4.3 to − 2.5	< 0.001	2008–2019	− 5.7*	− 6.3 to − 5.2	< 0.001				
Female	2001–2006	− 0.6	− 2.2 to 1.1	0.480	2006–2019	− 3.6*	− 4.0 to − 3.2	< 0.001	2017–2019	− 4.1*	− 6.8 to − 1.2	0.009
South												
Male	2001–2004	–0.2	–2.5 to 2.2	0.867	2004–2013	− 4.0*	–4.5 to –3.4	< 0.001	2013–2019	− 7.1*	− 8.1 to − 6.2	< 0.001
Female	2001–2005	2.0*	0.4 to 3.7	0.020	2005–2012	− 1.5*	− 2.3 to − 0.6	0.003	2012–2019	− 5.1*	− 5.8 to − 4.4	< 0.001
Northeast												
Male	2001–2008	− 2.3*	− 2.8 to − 1.8	< 0.001	2001–2009	− 0.2	− 1.1 to 0.7	0.675				
Female	2008–2019	− 4.3*	− 4.5 to − 3.9	< 0.001	2009–2019	− 3.6*	− 4.3 to − 2.9	< 0.001				
Midwest												
Male	2001–2009	− 1.4*	− 2.2 to − 0.5	0.005	2009–2019	− 4.4*	− 5.1 to − 3.7	< 0.001				
Female	2001–2013	0.1	− 0.5 to 0.7	0.758	2013–2019	− 5.7*	− 7.6 to − 3.8	< 0.001				
Histology												
Small cell												
Male	2001–2008	− 2.6*	− 3.7 to − 1.5	< 0.001	2008–2019	− 5.9*	− 6.5 to − 5.2	< 0.001				
Female	2001–2009	− 0.5	− 1.2 to 0.2	0.165	2009–2019	− 5.0*	− 5.6 to − 4.5	< 0.001				
Adenocarcinoma												
Male	2001–2014	− 0.1	− 0.6 to 0.4	0.759	2014–2019	− 6.8*	− 9.0 to − 4.6	< 0.001				
Female	2001–2013	2.4*	1.8 to 2.9	< 0.001	2013–2019	− 6.1*	− 7.7 to − 4.6	< 0.001				
Squamous cell												
Male	2001–2013	− 2.0*	− 2.4 to − 1.6	< 0.001	2013–2019	− 7.7*	− 9.1 to − 6.3	< 0.001				
Female	2001–2010	0.9	− 0.6 to 2.4	0.236	2010–2019	− 6.0*	− 7.6 to − 4.4	< 0.001				

*APC* annual percentage change. *AIAN* Non-Hispanic American Indian and Alaska Native; *API* non-Hispanic Asian or Pacific Islander; *CI* confidence interval; *NHB* non-Hispanic black; *NHW* non-Hispanic white.

*Indicates a significant change of APCs.

**Figure 8:**
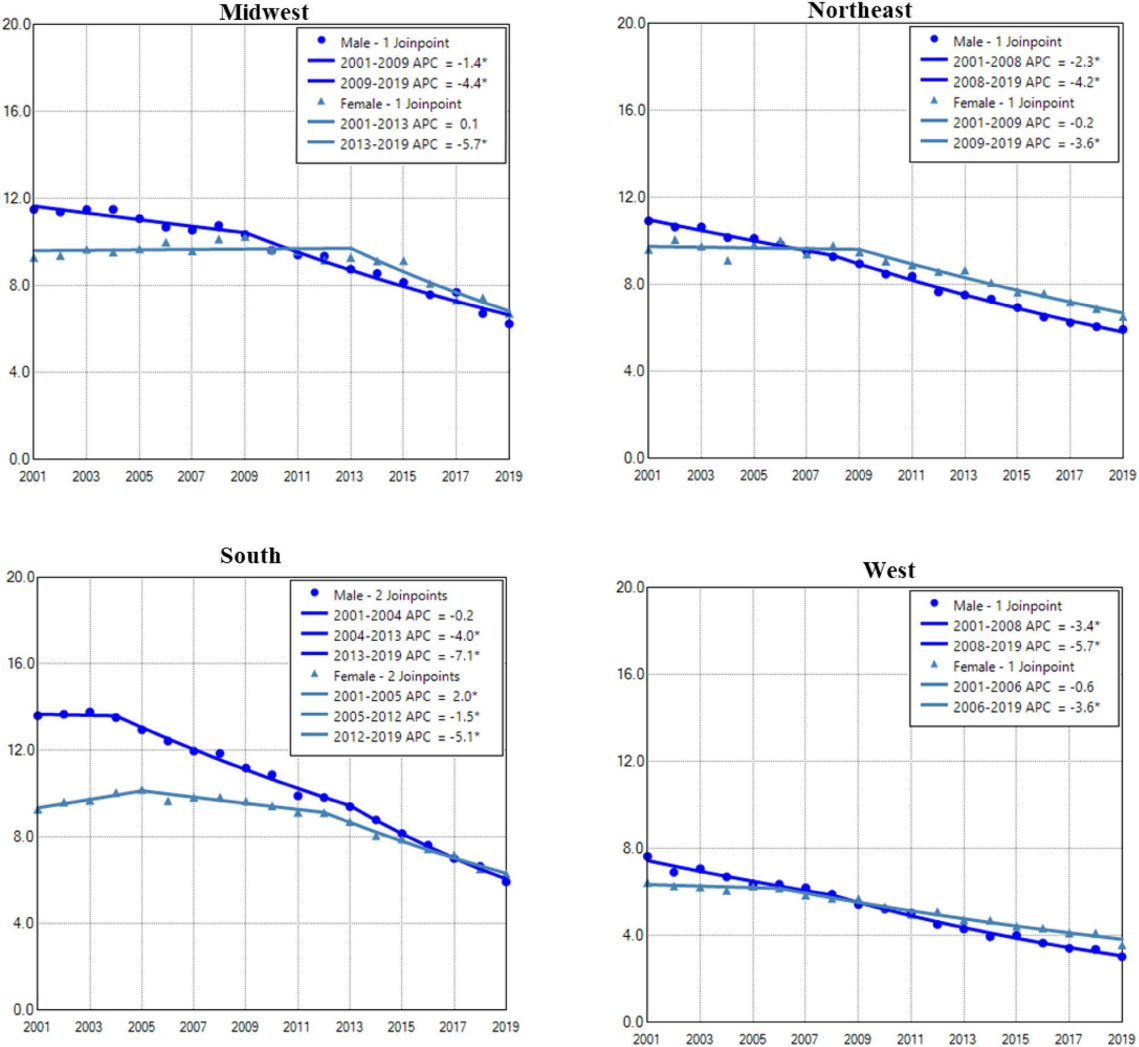
Temporal change of age adjusted incidence of lung cancer in males and females aged 0–54 years by region during 2001–2019. AAIR, age adjusted incidence rate; APC: annual percentage change. *Indicates a significant change in APC.

Among all regions, the West region had the lowest incidence in both males and females in the entire period. Females had higher lung cancer incidences than males in all regions in recent years. (Fig. 8 and Supplemental Data Figure S6). Males in all regions had continuous and faster decreases in lung cancer incidence in nearly the entire study period, whereas females in all regions showed declined incidences in recent years (Fig. 8 and Table 4).

**Figure 9:**
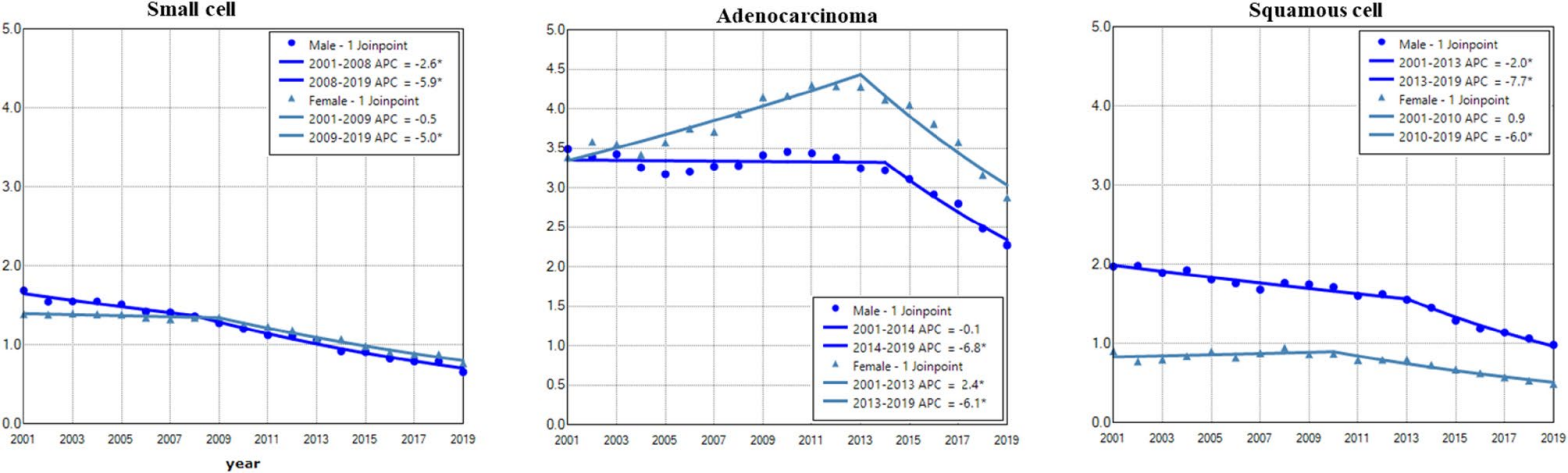
Temporal change of age adjusted incidence of lung cancer in males and females aged 0–54 years by histological type during 2001–2019. AAIR: age adjusted incidence rate; APC: annual percentage change. *Indicates a significant change in APC.

Among major histological types, adenocarcinoma had a much higher incidence in females during the entire study period, and small cell had higher incidences in females only between 2009 and 2019. Squamous cell incidence was higher in males than in females during 2001–2019 ((Fig. 9 and Supplemental Data Figure S7). Temporally, males had faster and continuous decreases in incidences of small and squamous cells during the entire period, but a decrease in adenocarcinoma incidence only during 2013–2019. Females had a slower decrease in incidences of these histological types in recent years (Fig. 9 and Table 4).

## Discussion

Using the database covering the entire US population, we examined the gender disparity in lung cancer incidence during 2001–2019. Our results revealed that the proportion of newly diagnosed lung cancer in females increased from 43.9% in 2001 to 49.3% in 2019. Females accounted for 51.7% of those aged 0–39 and 51.3% of adenocarcinoma. Males had a continuous and faster decline in lung cancer incidences during the entire study period, while females displayed an increased incidence during 2001–2006 and then a slower decline after that. Gender disparities in lung cancer incidence were observed among age groups, races, regions, and histological types. Among those aged 0–54 years, overall lung cancer incidence still displayed a faster decline in males in the entire study period but stabilized in females during 2001–2009 and declined during 2009–2019. In recent years, females began to display higher incidence rates of lung cancer than males in all races except NHB, all regions, and adenocarcinoma and small cell, but not squamous cell. NHB females aged 0–54 even had a faster rate of decreased incidence than males during 2013–2019. Furthermore, API females aged 0–54 demonstrated a slowly increased trend of lung cancer incidence during the study period.

This study revealed that the gender difference remained in overall lung cancer incidence. Males had a faster and continuous decrease in lung cancer incidence with APCs of − 1.3 during 2001–2007 and − 2.7 during 2007–2019. Lung cancer incidence in females increased (APC of 0.8) between 2001 and 2006 and decreased (APC of − 1.2) between 2006 and 2019. Though males had higher incidence rates of lung cancer than females during the entire study period, the female to male rate ratio and the proportion of female patients increased during 2001–2019. Like our findings, several previous studies reported a similar pattern of gender disparity in lung cancer in earlier years. A very recent study revealed that the incidence rate of lung cancer decreased by an APC of − 2.6 in men and an APC of − 1.1 in women since 2006–2007^1^. Lu et al.^6^ reported that males displayed a continuous and steeper decline of lung cancer incidence during 1982–2015. Females had an increased trend during 1973–2007 and then a decreased trend during 2007–2015. Using the SEER 9 database, an earlier study reported a decline in lung cancer incidence in males from 1988 to 2015 but an increased incidence in females between 1988 and 2006 and then a decreased trend from 2006 to 2015^7^. Other studies reported a gender disparity in lung cancer incidence in the earlier years ^2–5^. This and other studies indicate that males had a continuously faster decrease in lung cancer incidence in the past three decades, and females had a slower decline only since 2006–2007, leading to a diminishing difference in lung cancer incidence between gender.

A notable gender disparity in lung cancer incidence was observed in those aged 0–54 years. Lung cancer incidence continuously decreased faster in males during 2001–2019 but stabilized between 2001 and 2009 and then declined in females during 2009–2019. Remarkably, incidence rates were higher in females aged 0–39 years during 2001–2019, and aged 40–54 years during 2012–2019. In this young age group, higher incidences of lung cancer in females than males were observed in recent years in all races except NHB, in all geographic regions, and in adenocarcinoma and small cell. NHB females had a lower lung cancer incidence than males in the entire period. The female to male rate ratio even showed a decreased trend in NHB in recent years because females had a faster rate of declined incidence rates. The incidence of squamous cell lung cancer was much lower in females than in males during the entire period. The female to male rate ratio of squamous cell was stabilized at around 0.5 in the past decade. Similarly, a previous study observed that females 30–54 years old had higher incidence rates of lung cancer than males only in white and Hispanics during 2010–2014^8^. A higher incidence of lung cancer in females than in males aged 50 years or younger have been reported in earlier years^3^. However, another study showed that young adults aged 20 to 39 years had a higher incidence in females during 1995–2011, then a lower incidence in females from 2011–2015^7^. The gender disparity in young patients implies distinctive causes of lung cancer in man and females.

Smoking is estimated to cause over 80% of lung cancer in the US^11^. The higher overall incidence of lung cancer in males than in females has been associated with their smoking patterns. Historically, males have higher prevalence of smoking than females at any time points, more cigarettes per day^8,12^, and higher rates of the use of tobacco related products^12^. Men were more likely to smoke more harmful non-menthol cigarettes^13,14^. The decreased smoking prevalence in the past decades attributes to reduced incidences of lung cancer and other cigarette related cancers. The gender disparity in the temporal change in lung cancer incidence is partially explained by the fact that women started cigarette smoking in large numbers later and were slower to quit^15–17^.

The gender difference in lung cancer incidence is also because non-smoking lung cancer occurs disproportionally higher in women^18–22^. The rate of diagnosis of lung cancer in non-smokers has doubled in the last decade^23^. Many non-smoking risk factors have been implicated in lung cancer development, including lung carcinogens, indoor cooking fumes and secondhand smoke^24,25^, human papillomavirus (HPV)^26^. Lung cancer in young ages is often correlated with genetic risk or family history^25^. Females have been reported to carry susceptibility polymorphisms and loci, and higher frequency of mutations in critical driver genes, such as TP53 and the KRAS^27–29^.

In addition to genetic predisposition and natural history, lung cancer incidence is also associated with socioeconomic status, the health care system, and smoking control programs. Socioeconomic factors, such as income, education, occupation, and residing place, are strongly associated with healthy lifestyles, awareness of health care, cancer screening, detection, treatment, and survival^12,30^. Compared to males, females had a higher rate of healthcare access and higher medical care service utilization^31^. States differ widely in tobacco control programs and policies^32,33^. Differences in these factors may contribute to regional variations of lung cancer incidence^12,34,35^.

Complex interactions between smoking and other risk factors may explain the observed gender disparity among histopathological types^36–39^. This study corroborated that adenocarcinoma was the most common histologic type in both males and females. During 2001–2015, its incidence significantly increased in both males and females, with a slightly more rapid rate in females. The increased trend of adenocarcinoma incidence in earlier years was reported in previous studies^2,6^, which has been related to more smoking of low-tar filter cigarettes and improvements in the classification of histologic type^2^. Moreover, adenocarcinoma displayed a less rapid decline in comparison with squamous or small cell in responses to smoking cessation^40,41^. Due to the difference in smoking patterns and exposure to other risk factors, females were more vulnerable to adenocarcinoma^39^. Furthermore, females are more likely to be diagnosed at a younger age and with adenocarcinoma^2,8,42^. This study indicated that females aged 0–54 years had a higher incidence of adenocarcinoma than males during the entire study period. Squamous and small cell lung cancers are strongly correlated with heavy smoking^43,44^ and small cell lung cancer possesses a distinct molecular pathogenesis^44^.

This and other studies observed that NHB males had the highest incidence of lung cancer than other races^5,6^, but Hispanics and API males and females had a lower incidence^45,46^. The racial disparity in lung cancer incidence is correlated with the varied prevalence of smoking among races^47^. The higher incidence in NHB males is also correlated with their lower smoking cessation rates^48^, the larger proportion in health care non-expansion states^30^, and the elevated odds of missing lung cancer screening appointments^49^. NHB females aged 0–54 years showed a lower incidence of lung cancer than males during the entire study period. However, females in other races displayed higher incidences than males in recent years. Notably, NHB females in this age group had a faster decline in lung cancer incidence than males during 2013–2019, and both NHB males and females had a more rapid decline than other races. Similarly, a previous study showed that incidence rates declined in age groups 30–49 years in both blacks and whites, with the decline considerably larger in blacks before 2016^50^. The fast decrease in black males and females has been associated with their fast decrease in smoking prevalence^50^. It will be of clinical significance to unravel what other factors contribute to the more rapid reduction in lung cancer in NHB.

Limitations of this study include retrospective and observational designs and descriptive findings. Though the database allows calculating age adjusted incidence rates of lung cancer, much information is not available in the database. These cases have no detailed clinicopathological data, such as tumor size, T, N and M stages, and treatments. There is no data on smoking and other risk factors, such as diet, exercise, and family history. Socioeconomic statuses and health insurance data are also not available. The database lacks survival data for these patients. The strength of this study is that we utilize the database, which includes all lung cancer patients diagnosed in the US and in the most updated years.

## Conclusions

Overall lung cancer incidence continues to decrease with a gender disparity. Females begin to display a higher incidence of lung cancer than males at young ages. Continuous anti-smoking programs plus mitigation of other related risk factors curbing are necessary to further reduce the lung cancer burden in the United States.

### Supplementary Information

Supplementary Information.

## Data Availability

All data was obtained from the publicly available NPCR and SEER database (https://www.cdc.gov/cancer/uscs/public-use/index.htm with the username of 25,367-Nov2021).
