# Supplementary material for: Gender disparities in lung cancer incidence in the United States during 2001–2019
Source: Sci Rep. 2023 Aug 3;13:12581. doi: 10.1038/s41598-023-39440-8 (PMC10400573; doi:10.1038/s41598-023-39440-8)

**Supplemental data**

**Table S1.** Case number and proportion of lung cancer in males and females during 2001-2019.

| Year | Total | Males  n(%) | Female  n(5) |
| --- | --- | --- | --- |
| 2001 | 195,939 | 109,982(56.1) | 85,957(43.9) |
| 2002 | 198,954 | 110,710(55.6) | 88,244(44.4) |
| 2003 | 205,126 | 113,965(55.6) | 91,161(44.4) |
| 2004 | 206,287 | 113,844(55.2) | 92,443(44.8) |
| 2005 | 210,777 | 115,095(54.6) | 95,682(45.4) |
| 2006 | 211,828 | 114,898(54.2) | 96,930(45.8) |
| 2007 | 213,532 | 115,449(54.1) | 98,083(45.9) |
| 2008 | 216,462 | 116,390(53.8) | 100,072(46.2) |
| 2009 | 217,401 | 116,387(53.5) | 101,014(46.5) |
| 2010 | 214,637 | 114,564(53.4) | 100,073(46.6) |
| 2011 | 215,055 | 114,516(53.2) | 100,539(46.8) |
| 2012 | 217,981 | 115,249(52.9) | 102,732(47.1) |
| 2013 | 219,583 | 115,677(52.7) | 103,906(47.3) |
| 2014 | 221,587 | 116,371(52.5) | 105,216(47.5) |
| 2015 | 224,815 | 116,957(52) | 107,858(48) |
| 2016 | 224,995 | 116,594(51.8) | 108,401(48.2) |
| 2017 | 226,950 | 116,628(51.4) | 110,322(48.6) |
| 2018 | 223,429 | 113,711(50.9) | 109,718(49.1) |
| 2019 | 221,097 | 112,003(50.7) | 109,094(49.3) |
| Total | 4,086,435 | 2,178,990(53.3) | 1,907,445(46.7) |

**Figure S1.** Female to male of rate ratio among age groups during 2001-2019.


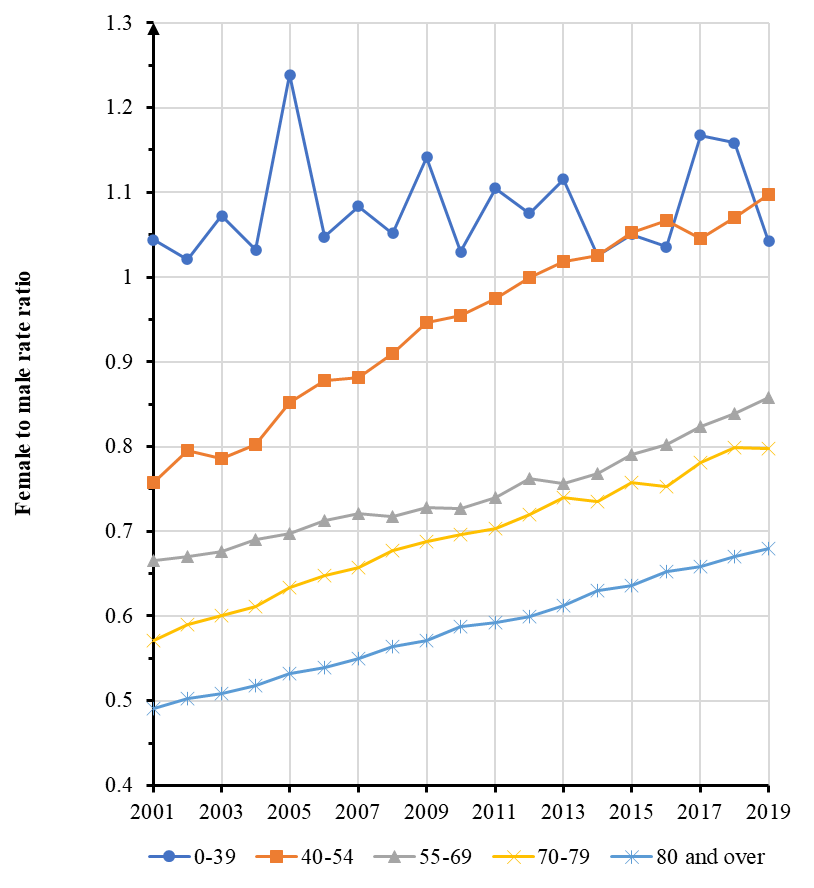


**Figure S2.** Female to male of rate ratio among races during 2001-2019. AIAN: non-Hispanic American Indian and Alaska Native; API: non-Hispanic Asian or Pacific Islander; NHB: non-Hispanic black; NHW: non-Hispanic white.


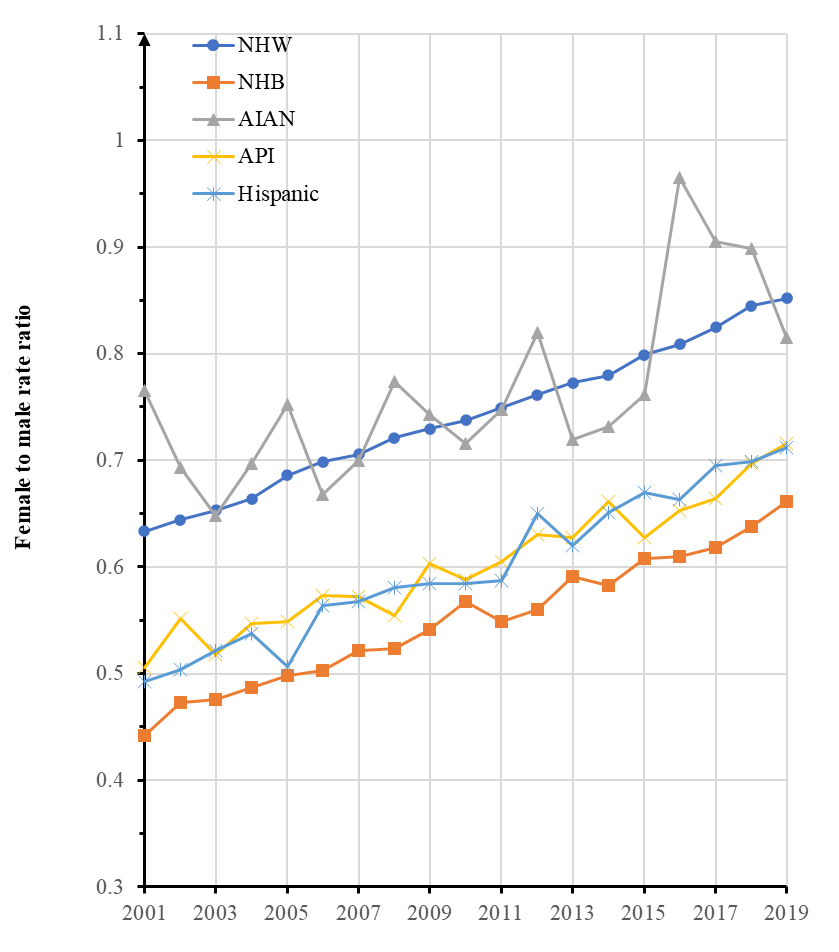


**Figure S3.** Female to male of rate ratio among regions during 2001-2019.


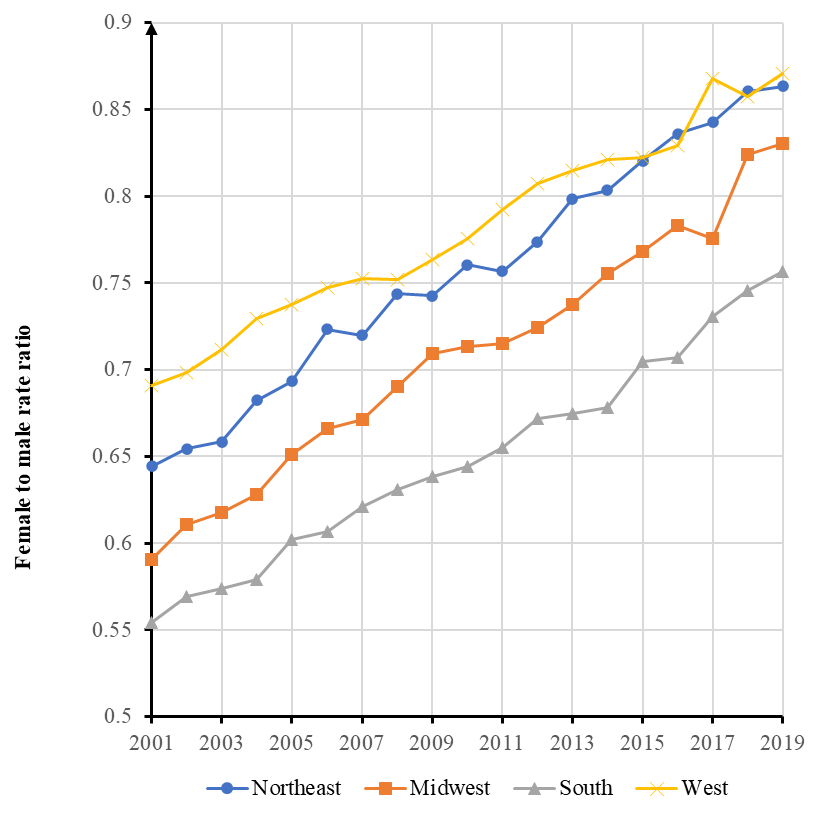


**Figure S4.** Female to male rate ratio among major histological types during 2001-2019.


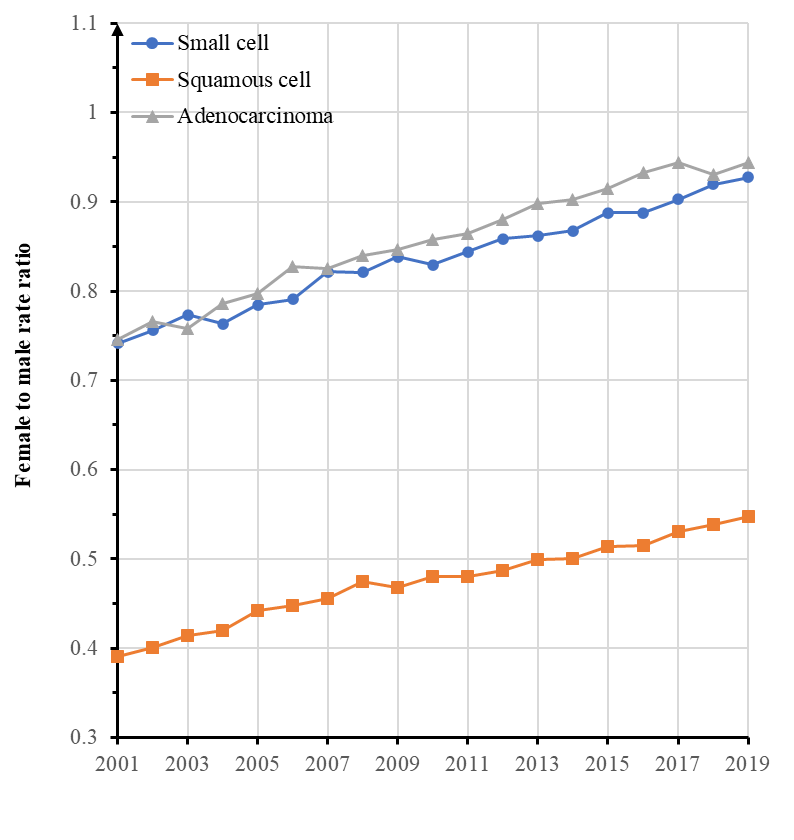


**Figure S5.** Female to male of rate ratio among races aged 0-54 years during 2001-2019. AIAN: non-Hispanic American Indian and Alaska Native; API: non-Hispanic Asian or Pacific Islander; NHB: non-Hispanic black; NHW: non-Hispanic white.


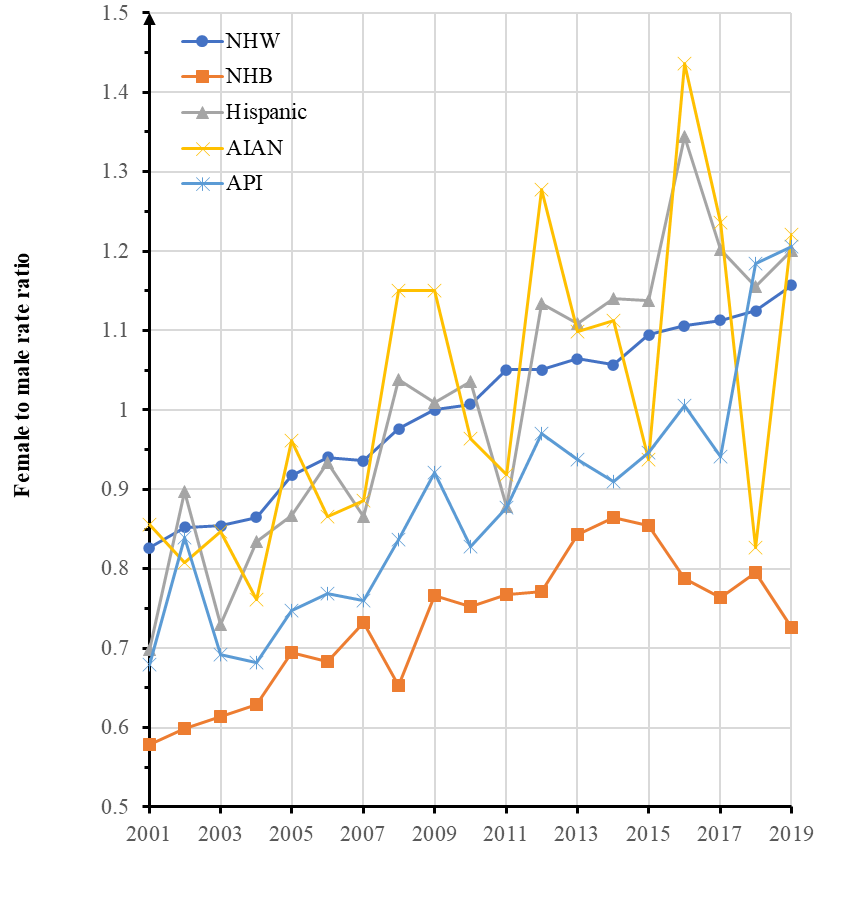


**Figure S6.** Female to male of rate ratio among regions aged 0-54 years during 2001-2019.


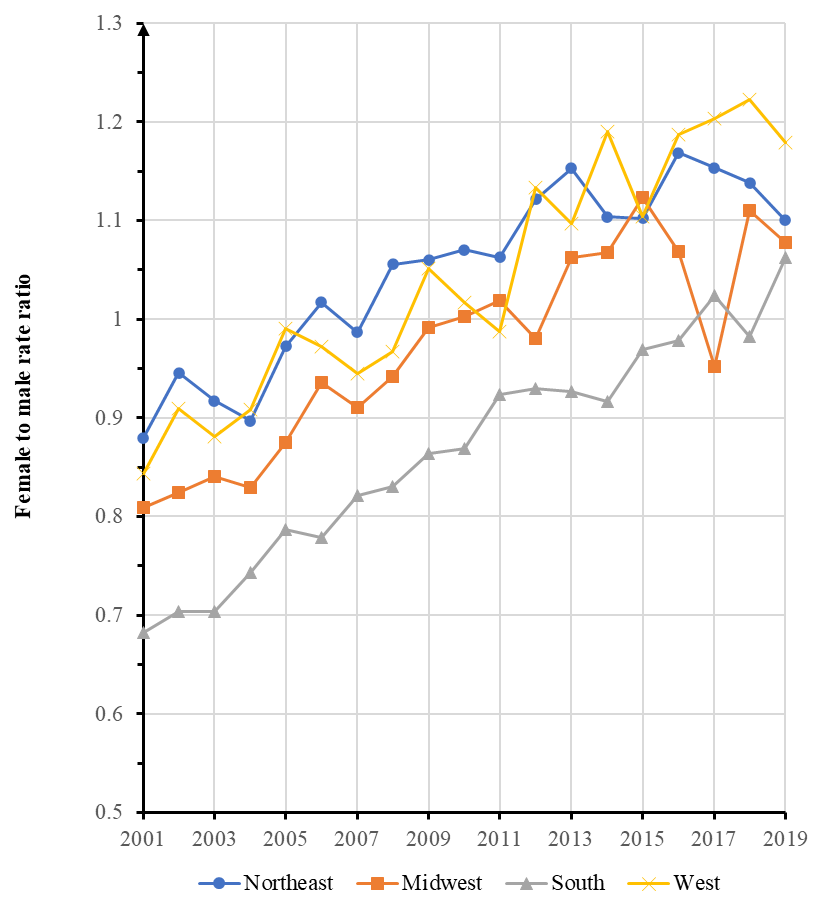


**Figure S7.** Female to male rate ratio among histological types aged 0-54 years during 2001-2019.


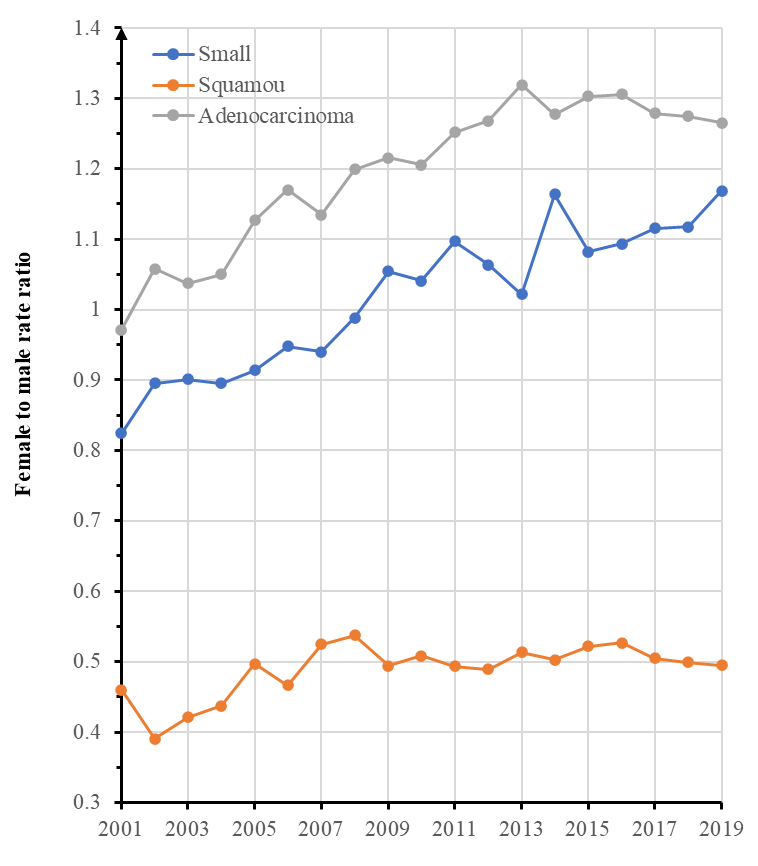

Supplement: Supplementary file 1 — Supplementary Information. [file 41598_2023_39440_MOESM1_ESM.docx]
